# Network Self-Organization Explains the Statistics and Dynamics of Synaptic Connection Strengths in Cortex

**DOI:** 10.1371/journal.pcbi.1002848

**Published:** 2013-01-03

**Authors:** Pengsheng Zheng, Christos Dimitrakakis, Jochen Triesch

**Affiliations:** 1 Frankfurt Institute for Advanced Studies, Frankfurt am Main, Germany; 2 Ėcole Polytechnique Fédérale de Lausanne (EPFL), Lausanne, Switzerland; Indiana University, United States of America

## Abstract

The information processing abilities of neural circuits arise from their synaptic connection patterns. Understanding the laws governing these connectivity patterns is essential for understanding brain function. The overall distribution of synaptic strengths of local excitatory connections in cortex and hippocampus is long-tailed, exhibiting a small number of synaptic connections of very large efficacy. At the same time, new synaptic connections are constantly being created and individual synaptic connection strengths show substantial fluctuations across time. It remains unclear through what mechanisms these properties of neural circuits arise and how they contribute to learning and memory. In this study we show that fundamental characteristics of excitatory synaptic connections in cortex and hippocampus can be explained as a consequence of self-organization in a recurrent network combining spike-timing-dependent plasticity (STDP), structural plasticity and different forms of homeostatic plasticity. In the network, associative synaptic plasticity in the form of STDP induces a rich-get-richer dynamics among synapses, while homeostatic mechanisms induce competition. Under distinctly different initial conditions, the ensuing self-organization produces long-tailed synaptic strength distributions matching experimental findings. We show that this self-organization can take place with a purely additive STDP mechanism and that multiplicative weight dynamics emerge as a consequence of network interactions. The observed patterns of fluctuation of synaptic strengths, including elimination and generation of synaptic connections and long-term persistence of strong connections, are consistent with the dynamics of dendritic spines found in rat hippocampus. Beyond this, the model predicts an approximately power-law scaling of the lifetimes of newly established synaptic connection strengths during development. Our results suggest that the combined action of multiple forms of neuronal plasticity plays an essential role in the formation and maintenance of cortical circuits.

## Author Summary

The computations that brain circuits can perform depend on their wiring. While a wiring diagram is still out of reach for major brain structures such as the neocortex and hippocampus, data on the overall distribution of synaptic connection strengths and the temporal fluctuations of individual synapses have recently become available. Specifically, there exists a small population of very strong and stable synaptic connections, which may form the physiological substrate of life-long memories. This population coexists with a big and ever changing population of much smaller and strongly fluctuating synaptic connections. So far it has remained unclear how these properties of networks in neocortex and hippocampus arise. Here we present a computational model that explains these fundamental properties of neural circuits as a consequence of network self-organization resulting from the combined action of different forms of neuronal plasticity. This self-organization is driven by a rich-get-richer effect induced by an associative synaptic learning mechanism which is kept in check by several homeostatic plasticity mechanisms stabilizing the network. The model highlights the role of self-organization in the formation of brain circuits and parsimoniously explains a range of recent findings about their fundamental properties.

## Introduction

The computations performed by cortical circuits depend on their detailed patterns of synaptic connection strengths. While the gross patterning of connections across different cortical layers has been well described in some cases [1], [2], the detailed connectivity structure between groups of cells and its relation to information processing have been notoriously difficult to investigate [3]. This detailed structure could either be largely random – the product of somewhat arbitrary growth processes, or it could be highly organized. On the one hand, randomly structured networks have been shown to possess powerful computational properties [4]–[6] and they are easy to generate. On the other hand, a precise non-random organization could be the product of network self-organization, where network structure determines neural activity patterns and activity patterns in turn shape network structure through plasticity mechanisms. At the macroscopic and mesoscopic scales, models based on self-organization have already explained fundamental features of brain networks. Examples are the formation of topographic mappings [7] or properties of orientation preference maps in primary visual cortex [8], [9]. Here we show that fundamental aspects of the microscopic structure of cortical networks can also be understood as the product of self-organization.

Self-organization typically relies on a combination of self-reinforcing (positive feedback) processes that are combined with a competition for limited resources. In the context of Neuroscience, an example of a self-reinforcing process may be that correlated firing of two groups of neurons may strengthen synaptic connections between them according to Hebb's postulate of synaptic plasticity, while the strengthened connections will in turn amplify the correlated firing of the neurons. An example for competition for a limited resource may be a synaptic scaling mechanism that limits the sum of a neuron's synaptic efficacies such that one synapse can only grow at the expense of others. The combination of self-reinforcing mechanisms with limited resources often gives rise to the formation of structural patterns, which may or may not have specific functional advantages. Here, we will offer an explanation for fundamental aspects of the fluctuations of synaptic strength and the distribution of synaptic efficacies based on self-organization.

Specifically, recent evidence shows that the distribution of synaptic efficacies is highly skewed [10], [11], having an approximately lognormal distribution [12]–[14]. Only around 20% of synapses are responsible for 50% of total synaptic weight. Importantly, synaptic contacts are constantly being created and destroyed and sizes of dendritic spines are fluctuating over time scales of hours and days [14], [15]. In the face of this highly dynamic network structure, stable long-term memories are thought to be based on subsets of synapses with long life times [16], [17], which may also be comparatively strong [16]. In line with this, the daily fluctuations of dendritic spine sizes, which are closely related to synaptic efficacies, are such that weak synapses can change their size by as much as a factor of 6, while strong synapses are much more stable [15].

To investigate whether and how these properties can arise from self-organization induced by neuronal plasticity mechanisms, we have developed a self-organizing recurrent network (SORN) model. It extends a previous model [18], and consists of noisy binary threshold spiking neurons (80% excitatory and 20% inhibitory) and uses five different forms of plasticity (see Materials and Methods for details). Connections between excitatory neurons are subject to an additive spike-timing dependent plasticity (STDP) rule that changes synaptic strength in a temporally asymmetric causal fashion as reported experimentally [19], [20]. A synaptic normalization mechanism keeps the sum of all excitatory weights to a neuron constant and models classic findings on multiplicative synaptic scaling of synaptic efficacies [21], [22]. An intrinsic plasticity mechanism adjusts the firing thresholds of excitatory neurons to maintain a low average firing rate. This mechanism models homeostatic changes in neuronal excitability through modification of voltage gated ion channels observed experimentally [23], [24]. Connections from inhibitory neurons onto excitatory neurons are subject to an inhibitory spike-timing dependent plasticity (iSTDP) rule that balances the amount of excitatory and inhibitory drive that the excitatory neurons receive as reported in recent studies [25]–[27]. Finally, a structural plasticity rule generates new synaptic connections between excitatory cells at a small rate. This models the constant generation of new synaptic contacts observed in cortex and hippocampus [15], [28].

## Results

### SORN produces lognormal-like weight distributions

**Figure 1:**
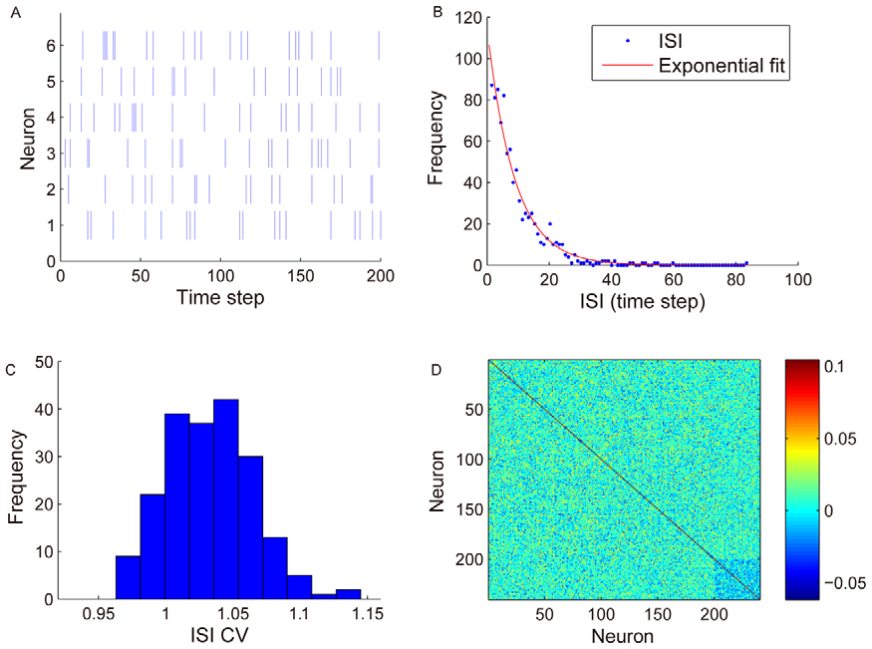
Irregular firing activity in the network around 10000 time step. A: spike trains of six randomly selected excitatory neurons during 200 time steps. B: example of an ISI distribution and exponential fit of a typical excitatory neuron. C: histogram of CV values of a network's excitatory units. D: correlations between all neurons. Neurons 201–240 are inhibitory. Network activities within the first 3000 steps are discarded to accommodate for a washout of the arbitrary initial state.

**Figure 2:**
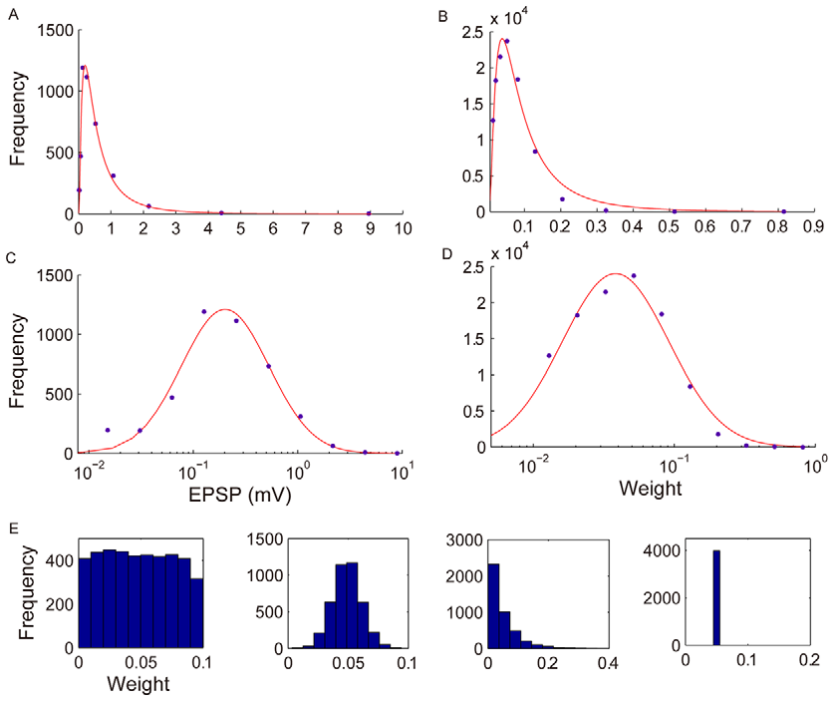
Distribution of synaptic weight strengths matches lognormal-like distribution of EPSPs in rat visual cortex. A: histogram of EPSP sizes from [12] and lognormal fit (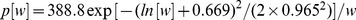). B: histogram of weight strengths in SORN at 10000th time step and lognormal fit (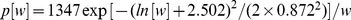). C, D: same as A, B but plotted with logarithmic scale on X-axis. E: examples of initial weight histograms drawn from different probability distributions that all lead to lognormal-like weight distributions. From left to right: uniform, Gaussian, exponential, all weights identical.

We simulated networks of 200 excitatory and 40 inhibitory neurons for 10,000 time steps and observed the resulting activity patterns (Fig. 1) and distributions of synaptic strength (Fig. 2). The network shows irregular activity patterns reminiscent of cortical recordings (Fig. 1A). Inter-spike interval (ISI) distributions are well fitted by an exponential function (Fig. 1B) and coefficient of variation (CV) values are close to one (Fig. 1C) as would be expected from a Poisson process. Neurons show only very weak correlations of their firing during this phase of network development (Fig. 1D).

To estimate the probability distribution governing excitatory-to-excitatory synaptic strengths we bin connection strengths and divide the number of occurrences in each bin by the bin size. The bin sizes are uniform on the log scale. To mimic experimental procedures [15], very small synapses (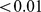) are excluded. Fig. 2A–D shows the distribution of synaptic connection strengths after 10,000 time steps and compares it to EPSP data from rat visual cortex [12]. With distinctly different initial conditions (Fig. 2E), the network faithfully develops a long-tailed distribution of connection strengths that is similar to the biological data (see Text S1 for details). Experimental data and model results are both well fit by lognormal distributions.

**Figure 3:**
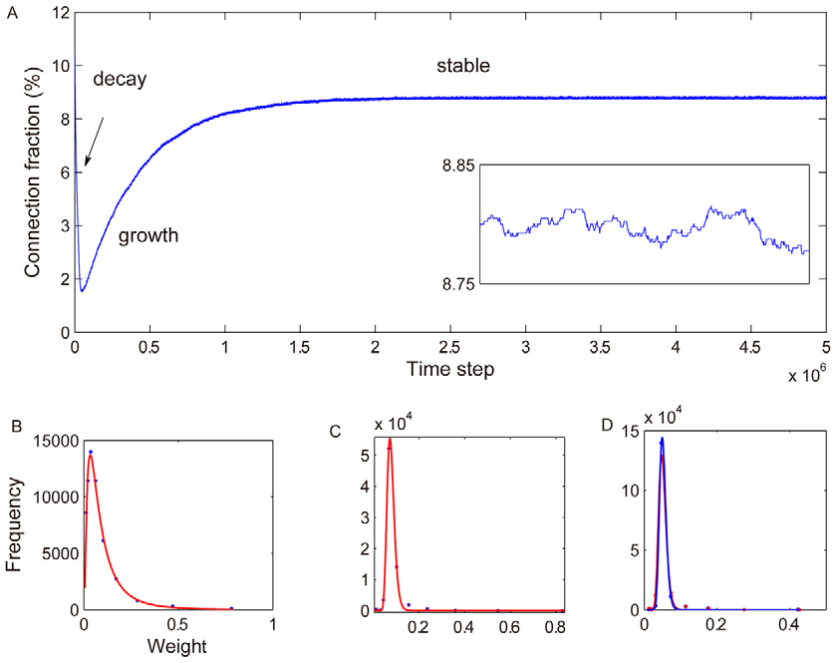
Long-term dynamics of the network. A: fraction of existing excitatory-to-excitatory connections recorded over 5 million time steps. The inset shows an enlargement of the last 1,000 steps. B: synaptic weight distribution recorded at 20,000th time step. C: synaptic weight distribution recorded at 500,000th time step. D: synaptic weights distributions recorded at 3,000,000th (blue dot) and 4,000,000th (red dot) time step. Blue and red curves in B–D are lognormal fits.

As the network evolves it goes through different phases (Fig. 3). The initial phase is characterized by a *decay* of connectivity, where a substantial fraction of the excitatory-to-excitatory synaptic weights get eliminated (Fig. 3A). In the subsequent *growth* phase, the network connectivity recovers through the integration of newly created synapses produced by the structural plasticity. Eventually, the degree of connectivity stabilizes and the network enters into a *stable* regime. Here, connectivity fluctuates very little (Fig. 3A inset). Newly created synapses tend to quickly disappear and there is a large stable backbone of connections with extremely long life times (as long as we simulated). The distribution of excitatory-to-excitatory connection strengths is lognormal-like throughout most of the network's evolution (Fig. 3B–D). (see Fig. S2 in Text S2 for more results with different parameters). An exception is the transition from the decay to the growth phase, where large deviations from the lognormal shape are observed (not shown). However, the distribution of synaptic weights maintains a long tail and a positive skewness throughout its development. The thresholds of the excitatory units in the network develop an approximately Gaussian distribution. In the stable regime of the network, this distribution is exhibiting only small fluctuations.

### Dynamics of synaptic efficacies in SORN matches experimental data

**Figure 4:**
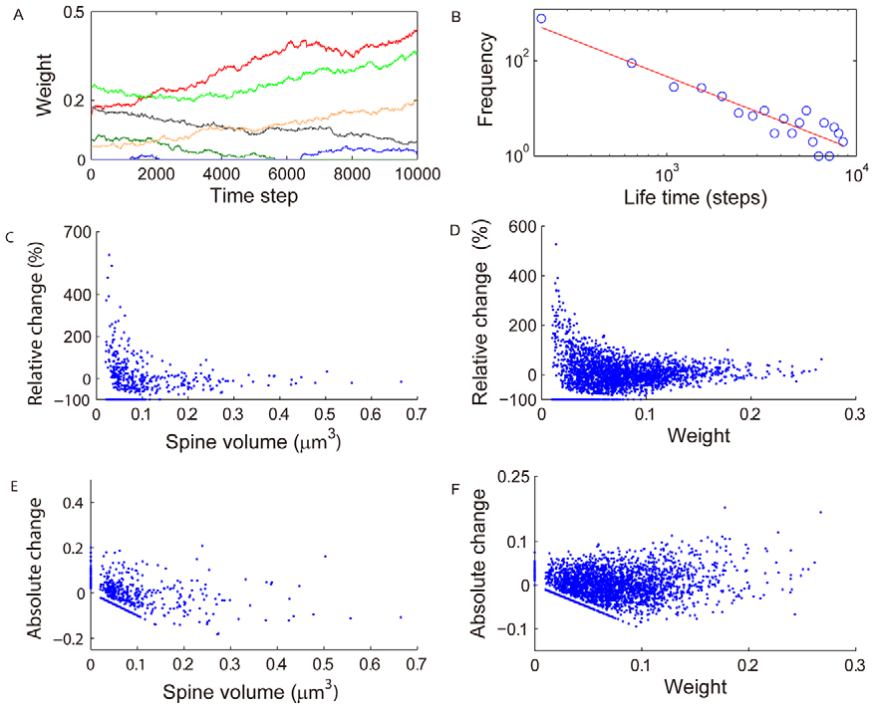
Distribution of synaptic weight changes matches distribution of spine volume changes in rat hippocampus. A: example traces of different synaptic weights. B: distribution of life times of newly created synapses matches a power law with exponent close to −3/2. C: distribution of relative spine volume changes across one day from [15]. D: distribution of synaptic weight changes in SORN over 3000 time steps. E, F: same as C, D but for absolute rather than relative changes in spine volume and synaptic weight, respectively.

As a next step, we assessed the dynamics of synaptic connection strengths in SORN. Fig. 4A shows traces of 6 synaptic connection weights as a function of time. The distribution of life times of newly created synapses is well described by a power law with an exponent close to −3/2 during this phase as expected for random walk behavior (Fig. 4B). We next compared the weight changes occurring in SORN over 3000 time steps with experimental data from time lapse imaging of dendritic spine sizes in rat hippocampus [15]. In both SORN and the experimental data, strong synapses are found to have comparatively small fluctuations (Fig. 4C–F). This is not a simple ceiling effect, since synaptic weights could, in principle, grow much larger than the typical values for very strong synapses we observe in the model, which lie between 0.2 and 0.3. There exists a small population of synaptic connections in both model and experimental data which decays completely (horizontal lines in Fig. 4C,D and oblique lines in Fig. 4E,F). The population of synapses clustered on the Y-axis in Fig. 4E,F represents newly established synaptic connections. The big fluctuations are mostly seen in decay phase and imply that the network is far from stability in this regime (see Fig. S6 in Text S2 for additional results with different parameters showing weight fluctuations during different phases of network evolution).

### Multiplicative dynamics despite additive STDP

**Figure 5:**
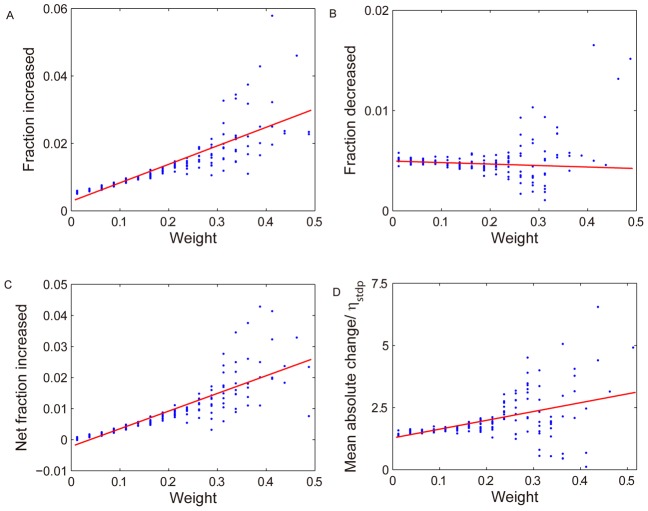
Rich-get-richer dynamics and linear growth of fluctuations. A: the average fraction of synaptic connections that increase due to STDP in one time step as a function of connection weight. B: same as A but for weight decreases due to STDP. C: average number of increased weights minus average number of decreased weights divided by total number of weights of this size. D: mean absolute change of synaptic weight due to STDP and synaptic scaling over 200 time steps.

To better understand the mechanism through which the network self-organizes its connectivity and dynamics, we examined how the strength of a synaptic connection influences its probability of undergoing further growth or decline. Among all the plasticity mechanisms, only STDP and synaptic normalization adjust the weights of EE connections. While synaptic normalization will only scale all incoming excitatory-to-excitatory connections linearly, STDP has the power to change the shape of the distribution of synaptic weights impinging onto a neuron. When we recorded the isolated effect of STDP, *i.e.* independently of the synaptic normalization, we found that over a large range of synaptic weight strengths, the expected increase in strength of a connection due to STDP grows approximately linearly with the strength of the synapse (Fig. 5A). The fraction of connections undergoing depression depends much less on connection weight (Fig. 5B). Thus, the net effect is that stronger synaptic connections have a higher chance to be potentiated by STDP establishing a rich-get-richer behavior (Fig. 5C). This mechanism is kept in check by the synaptic normalization mechanism, which scales weights in a multiplicative fashion. We estimated the mean absolute change of synaptic connection strengths due to STDP and synaptic normalization over 200 time step intervals during the initial 10,000 time steps. The mean absolute sizes of fluctuations grow roughly linearly with weight (Fig. 5D) as observed experimentally [14]. Note that this approximately linear dependence on weight strength occurs despite the additive STDP rule we are using and does not require a multiplicative STDP rule [12].

### Homeostatic plasticity mechanisms are essential for proper self-organization

With all forms of plasticity present, the network will show irregular firing activity and develop a lognormal-like weight distribution. These results are stable over a large range of parameter values (see Text S2 for details). To investigate the extent to which the different forms of plasticity contribute to these results, we performed simulations where we switched off individual plasticity mechanisms. When synaptic normalization is switched off, the network will show bursts of high activity separated by long periods of inactivity. As shown in Fig. 4, the network keeps eliminating synapses as a result of STDP. The structural plasticity counteracts this process. If we switch off the structural plasticity, a large number of neurons eventually lose all their postsynaptic targets. No lognormal-like weight distribution will emerge if one or both forms of plasticity are missing.

**Figure 6:**
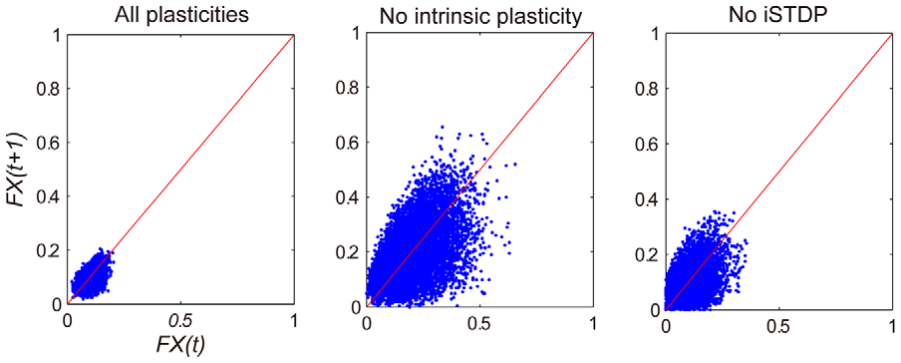
Different network activities observed with all plasticities and turning off intrinsic plasticity or iSTDP. 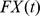
 denotes the fraction of excitatory neurons firing at time step 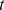. Red line is the identity line with 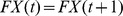. Network activities within the first 3000 steps are dismissed to accommodate for a washout of the arbitrary initial state.

Intrinsic plasticity and inhibitory STDP both try to maintain a low average firing rate of excitatory cells and both are important to keep healthy network dynamics. If both are switched off, some units will exhibit very high firing rates while others remain essentially silent and all the phenomena shown in Fig. 1–5 will disappear. To study the individual effects of intrinsic plasticity and iSTDP, Fig. 6 shows a scatter plot of the fraction of active excitatory units 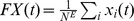 in subsequent time steps. With all plasticity mechanisms active, the network activity is confined within a small area. Activity never dies out or becomes very big. When either intrinsic plasticity or inhibitory STDP is switched off, the network activity exhibits big fluctuations and can temporarily die out completely. In certain parameter regimes the network may function without one or the other, but with both mechanisms being present, we obtain robust results over a large range of parameter values. We conclude that all five plasticity mechanisms are important for proper self-organization.

## Discussion

Understanding the structure and dynamics of neural circuits and reproducing them in neural network models remains a major challenge. Classic models of STDP have been shown to lead to physiologically unrealistic bimodal weight distributions under certain conditions [29]. This has lead to the proposal of a number of modifications to STDP rules to remedy the problem. Specifically, multiplicative STDP rules have received much interest recently [30], [31]. Here we have shown that an additive STDP rule when operating together with other plasticity mechanisms in a recurrent network is sufficient to explain both the statistics and fluctuations of synaptic connection strengths observed in cortex. Associative synaptic plasticity induces a rich-get-richer dynamics of synaptic weights, while homeostatic mechanisms induce competition. With distinctly different initial conditions, the ensuing self-organization faithfully develops Poisson-like irregular firing patterns, lognormal-like weight distributions and the characteristic pattern of fluctuations of synaptic strengths reminiscent of cortical recordings. Beyond this, our model predicts a power-law scaling of the lifetimes of newly established synaptic connections during development. Our results suggest that the statistics and dynamics of neural circuits are the product of network self-organization, and that the combined action of multiple forms of neuronal plasticity plays an essential role in the formation and maintenance of cortical circuits.

It is important, however, to also consider alternative explanations. One of the simplest ways to obtain lognormal distributions is by virtue of Gibrat's law, which was originally developed in Economics. It describes the growth of companies by random annual growth rates which are independent of the companies' sizes. This process by itself, when applied to the growth of synaptic connections, would predict that the variance of the synaptic weight distribution would grow without bounds, which is clearly at odds with biological reality. Adding a multiplicative normalization mechanism such as our synaptic normalization rule to Gibrat's proportionate growth process retains the development of a lognormal-like distribution while avoiding the problem of unbounded growth. However, this model does not reproduce the pattern of weight fluctuations observed experimentally. Furthermore, such a model is purely phenomenological and does not describe the mechanism that *causes* the synaptic fluctuations in the first place.

Similarly, the models proposed in [15] and [14] describe the fluctuations of synaptic weights as independent random walk processes, but do not explain what causes the synaptic fluctuations. In contrast, our model offers a *mechanistic* account that explains the patterns of weight fluctuations and the distribution of synaptic strength in terms of fundamental processes of neuronal plasticity in a recurrent network. This approach is consistent with the finding in [15] that the fluctuations of dendritic spine sizes seem to strongly depend on activity-driven synaptic plasticity. Specifically, they found strongly reduced fluctuations of spine sizes and fewer spine eliminations when inhibiting NMDA receptors with APV or MK-801. Interestingly, the generation of new spines was unaffected by this manipulations. This is consistent with our model's assumption that the generation of new spines occurs via a process of structural plasticity that is independent of activity-driven synaptic changes. A further advantage of our model is that it can also be used to derive predictions regarding the emerging network topology in terms of clustering, network motifs, etc. This topic is left for future work.

If our model is essentially correct, despite its very abstract formulation, then one should be able to replicate the present results in more realistic network models of spiking neurons. As a first step in this direction, we have constructed a version of the model using leaky-integrate-and-fire neurons with realistic parameter values. We have also adapted the plasticity mechanisms for this network. Initial explorations show that major features such as the lognormal-like weight distribution and the pattern of synaptic fluctuations can also be found in this less abstract network model. Future work will elaborate on these preliminary results.

Since the structure of cortical circuits determines the dynamics of neuronal activity, it also determines how information is encoded and propagated. The existence of a small number of very strong synaptic connections may greatly facilitate the highly reliable propagation of signals along pools of neurons [32]. In fact, SORN networks have previously been shown to spontaneously develop encoding strategies based on trajectories through their high-dimensional state space of unit activations [18]. In this work, the networks were fed with structured time series of input letters and were shown to learn internal representations of these input sequences that allowed large performance increases in prediction tasks. This was found to be due to the ongoing self-organization in the network driven by the network's plasticity mechanisms. They were shown to effectively increase the separation of network states belonging to different input conditions. More recently, we have found evidence that such networks may naturally self-organize to perform computations resembling Bayesian inference processes [33]. Further work is needed to better understand how the network's self-organization enables it to behave this way.

Many computational models of local cortical circuits assume random network structure [4]–[6], sometimes with distance-dependent or layer-dependent connection probabilities [34]. Such random network structure is at odds with recent evidence that changes to the connectivity structure such as the generation of stable new spines are associated with the formation of new memories [35]. Hence, we believe that the study of random networks where only connection statistics are matched to those in the brain, may be quite misleading when the goal is to understand processing in cortical circuits. Instead, self-organizing networks, which can faithfully develop brain-like activity and connectivity patterns, seem a much more promising subject of study.

## Materials and Methods

We use a SORN (self-organizing recurrent neural network) model [18] that uses noisy units, incorporates additional plasticity mechanisms, and receives no external input. The network is composed of 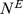 excitatory and 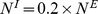 inhibitory threshold neurons connected through weighted synaptic connections. 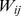 is the connection strength from neuron 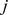 to neuron 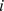. We distinguish connections from excitatory to excitatory neurons (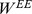), excitatory to inhibitory connections (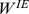) and inhibitory to excitatory connections (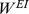). Connections between inhibitory neurons and self-connections of excitatory neurons are forbidden. The connections onto excitatory cells (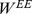 and 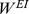) are subject to synaptic plasticity mechanisms described below. 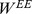 and 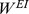 connections have sparse random initial connectivity with connection probabilities of 0.1 and 0.2, respectively. The 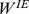 remain fixed at their random initial values. They have all-to-all topology and are drawn from the interval 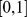 and subsequently normalized such that the incoming connections to an inhibitory neuron sum up to one: 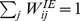.

The network's activity state, at a discrete time 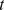, is given by the binary vectors 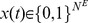 and 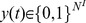 corresponding to the activity of the excitatory and inhibitory neurons, respectively. The evolution of the network state is described by:
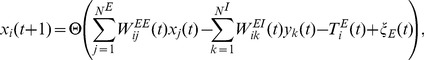
(1)

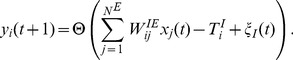
(2)


The 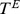 and 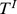 are threshold values for the excitatory and inhibitory neurons, respectively. They are initially drawn from a uniform distribution in the interval 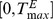 and 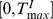. The Heaviside step function 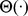 constrains the activation of the network at time 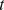 to a binary representation: a neuron fires if the total drive it receives is greater then its threshold, otherwise it stays silent. 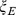 and 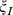 represent white Gaussian noise with 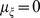 and 
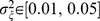
.

The time scale of a single iteration step in the model corresponds to typical membrane time constants and widths of spike-timing dependent plasticity (STDP) windows — lying roughly in the range of 10 to 20 ms. Note that in order to save computation time the homeostatic plasticity mechanisms described below are simulated to be much faster than in reality.

The network relies on several forms of plasticity: STDP of EE and EI connections, synaptic scaling and structural plasticity of EE connections, and intrinsic plasticity regulating the thresholds of excitatory neurons.

The set of 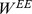 synapses adapts via a causal STDP rule that strengthens the synaptic weight 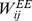 by a fixed amount 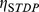 whenever neuron 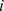 is active in the time step following activation of neuron 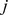. When neuron 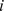 is active in the time step preceding activation of unit 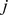, 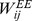 is weakened by the same amount (or set to zero if necessary to prevent it from becoming negative, which triggers synapse elimination):
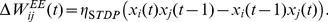
(3)


*Synaptic normalization* proportionally adjusts the values of incoming connections to an excitatory neuron at each time step so that they sum up to one:
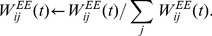
(4)This rule does not change the relative strengths of synapses established by STDP but regulates the total incoming drive a neuron receives and limits weight growth. It leads to a competition among excitatory-to-excitatory connections impinging onto the same neuron such that growth of some connections is compensated by the decay of others.

An *intrinsic plasticity* rule maintains a constant average firing rate in every neuron. To this end, a neuron that has just been active increases its threshold while an inactive neuron lowers its threshold by a small amount:
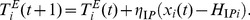
(5)where 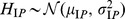 sets the target firing rate. For simplicity, one can also set the same target firing rate for all the excitatory neurons.

Note that the synaptic normalization and intrinsic plasticity mechanism operate faster in the model than they would in biological brains. This choice is warranted because of a separation of time scales and speeds up the simulations.

Compared to the original SORN model, we introduce two additional forms of plasticity. *Structural plasticity* adds new synaptic connections between excitatory cells to the network at a small rate, which balances the synapse elimination induced by STDP. With probability 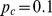 a new connection is added between a random pair of excitatory cells that are unconnected. The strength of this weight is set to 0.001.

*Inhibitory spike-timing dependent plasticity (iSTDP)* adjusts the weights from inhibitory to excitatory neurons to balance the amount of excitatory and inhibitory drive a neuron is receiving. If the inhibitory neuron spikes and the excitatory neuron remains silent in the subsequent time step (the inhibitory spike was “successful” in preventing the excitatory cell from spiking), the inhibitory weight is reduced by an amount 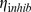 (or set to a small positive value of 0.001 if necessary to prevent it from being eliminated). If, however, the inhibitory neuron spikes and the excitatory neuron also spikes in the subsequent time step (the inhibitory spike was “unsuccessful” in preventing the excitatory cell from spiking), the inhibitory weight is increased by the larger amount 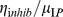. In all other cases the weight remains unchanged:
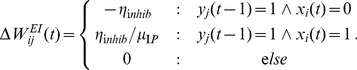
(6)


Equivalently, we can write:
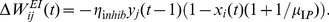
(7)Unless otherwise specified, the initial weights of 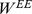, 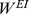 and 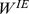 are drawn from a uniform distribution as shown in Fig. 2E, and the simulations are conducted using the following parameters. 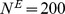, 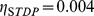, 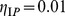, 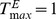, 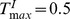, 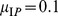, 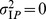, 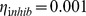, 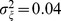.

## Supporting Information

Text S1 Comparison of SORN weight distribution to experimental data.
(PDF)

Text S2 Parameter robustness analysis and long-term dynamics.
(PDF)
