# Supplementary material for: Network Self-Organization Explains the Statistics and Dynamics of Synaptic Connection Strengths in Cortex
Source: PLoS Comput Biol. 2013 Jan 3;9(1):e1002848. doi: 10.1371/journal.pcbi.1002848 (PMC3536614; doi:10.1371/journal.pcbi.1002848)
Supplement: Text S1 — Comparison of SORN weight distribution to experimental data. (PDF) [file pcbi.1002848.s001.pdf]

Supporting Information Text S1

Comparison of SORN weight distribution to experimental data

We performed a statistical test to study the similarity between two distributions. In the simplest model, we assume that each synaptic weight w_k has been drawn independently from some unknown distribution P defined on (the Borel sets of) $[0, \infty)$. Consequently, the vector of all n weights is drawn from the product distribution: $\mathbf{w} \sim P^n$.

Since point-null hypothesis testing using p-values is in general misleading [1], we instead report a more informative statistic, which quantifies how likely it is that the two generating distributions are far apart. The first step is to search for the linear transformation minimizing the max-norm difference between the two empirical cdfs. We then use the Dvoretzky-Kiefer-Wolfowitz inequality to bound the gap between any empirical cdf and that of the distribution from which the sample was drawn. This gives high probability upper and lower bounds $\epsilon_{\max}$ and $\epsilon_{\min}$ on the distance ϵ between two unknown distributions, from which we have two samples. The result is shown in Figure S1, which succinctly quantifies their difference for varying error probability shown on the horizontal axis. For example, at the point $\delta = 10^{-1}$, we observe that, with probability at least 0.9, the distance between the generating distributions is at most 0.1304. On the other hand, there is only a small chance of 0.1 that their distance is less than 0.004. One may then conclude that, while the distributions are very similar, they are are not necessarily identical.

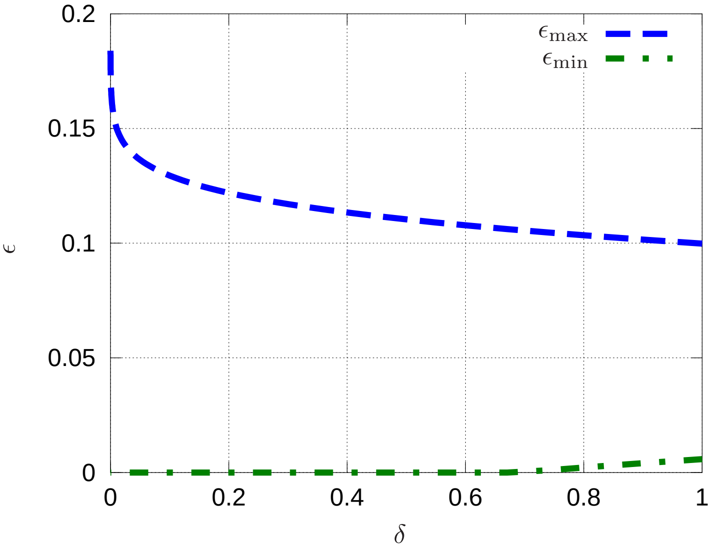

Fig. S1. Probabilistic bounds on the distance ϵ between the weight distributions of the experimental data and the SORN data. The horizontal axis is a bound on the probability that the distance ϵ is not within the given bounds, i.e. $\delta \geq \mathbb{P}(\epsilon \notin [\epsilon_{\min}, \epsilon_{\max}])$.

References

1. Berger JO, Sellke T (1987) Testing a point null hypothesis: the irreconcilability of p-values and evidence. Journal of the American Statistical Association 82:112-122.
