# Supplementary material for: Network Self-Organization Explains the Statistics and Dynamics of Synaptic Connection Strengths in Cortex
Source: PLoS Comput Biol. 2013 Jan 3;9(1):e1002848. doi: 10.1371/journal.pcbi.1002848 (PMC3536614; doi:10.1371/journal.pcbi.1002848)
Supplement: Text S2 — Parameter robustness analysis and long-term dynamics. (PDF) [file pcbi.1002848.s002.pdf]

Supporting Information S2

Parameter Robustness Analysis and Long-term Dynamics

Across a wide range of parameters, the network produces lognormal-like synaptic weight distributions and faithfully reproduces experimental data on synapse stability as a function of synaptic efficacy. Parameters tested: $N^E = [100, 1000]$, $\eta_{\text{STDP}} = [0.001, 0.005]$, $\eta_{\text{IP}} = [0.001, 0.2]$, $H_{\text{IP}} = [0.05, 0.2]$, $\eta_{\text{inhib}} = [0.005, 0.02]$, $\mu_{\text{IP}} = [0.08, 0.15]$, $\sigma^2_{\text{IP}} = [0.0001, 0.0015]$, $\sigma^2_{\xi} = [0.01, 0.1]$, $T^E_{max} = [0.5, 1]$, $T^I_{max} = [0.5, 1]$.

Example 1: Network with different firing rates and small noise

The following result are obtained with the following parameters: the initial weights of W^{EE}, W^{EI} and W^{IE} are drawn from a uniform distribution, $N^E = 200$, $\eta_{STDP} = 0.004$, $\eta_{IP} = 0.01$, $T^E_{max} = 1$, $T^I_{max} = 0.5$, $\mu_{\text{IP}} = 0.15$, $\sigma^2_{\text{IP}} = 0.001$, $\eta_{\text{inhib}} = 0.001$, $\sigma^2_{\xi} = 0.01$.

Compared to the main paper, this simulation has different target firing rates for excitatory neurons and smaller noise, but the network still faithfully reproduces all the key features as experimental data. According to our simulations, correlations of spike trains can become higher if we use a bigger mean firing rate μ_{IP} or smaller noise σ^2_{ξ}.

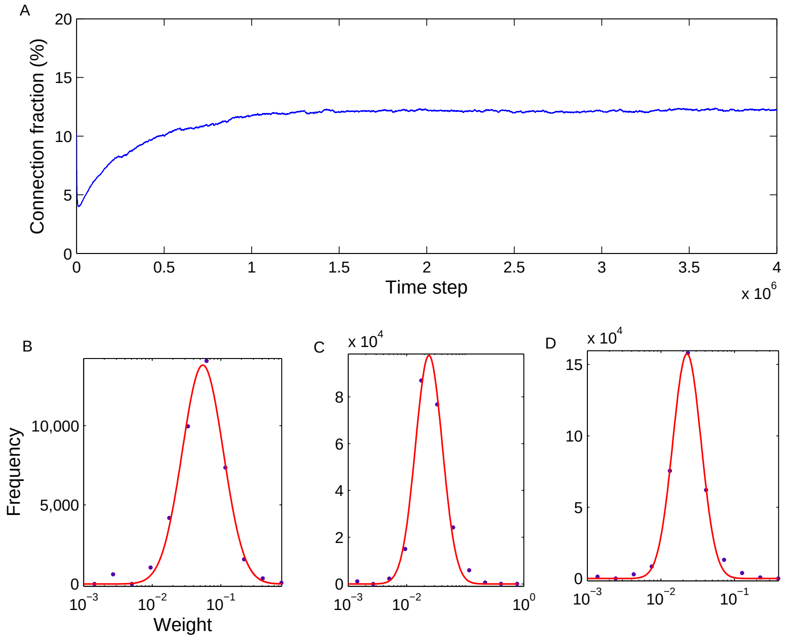

Fig. S2 Long-term dynamics of the network with different firing rate. A: fraction of existing excitatory-to-excitatory connections recorded over 4 million time steps. B: synaptic weight distribution recorded at 20,000th time step. C: synaptic weight distribution recorded at 500,000th time step. D: synaptic weight distributions recorded at 4,000,000th time step. Red curves in B–D are lognormal fits.

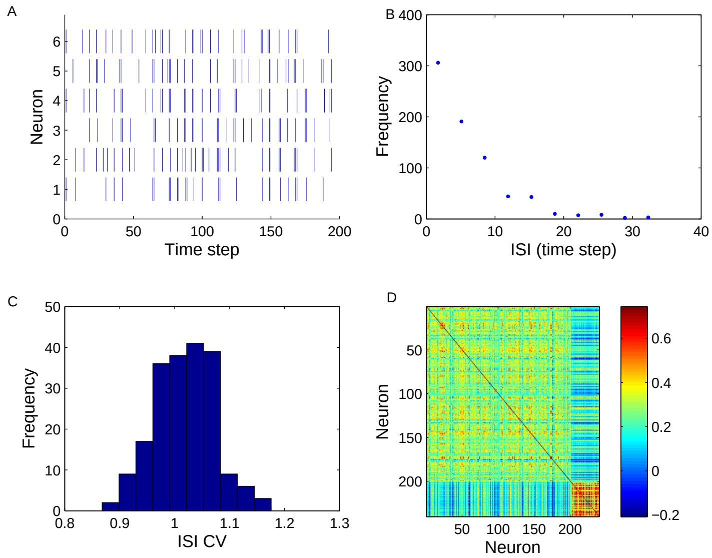

Fig. S3. Neuron activities at 20,000th time step. A: spike trains of six randomly selected excitatory neurons during 200 time steps. B: example of an ISI distribution. C: histogram of CV values of a network's excitatory units. D: correlations between all neurons.

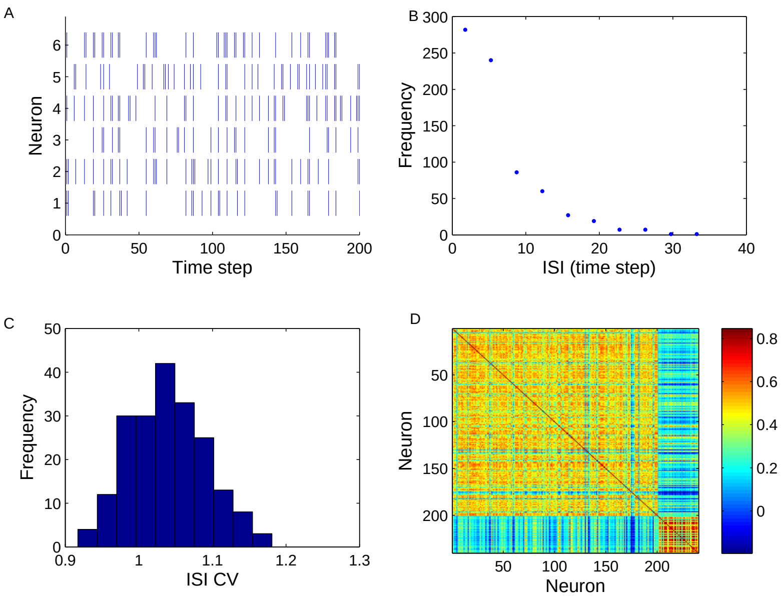

Fig. S4. Neuron activities at 500,000th time step. A: spike trains of six randomly selected excitatory neurons during 200 time steps. B: example of an ISI distribution. C: histogram of CV values of a network's excitatory units. D: correlations between all neurons.

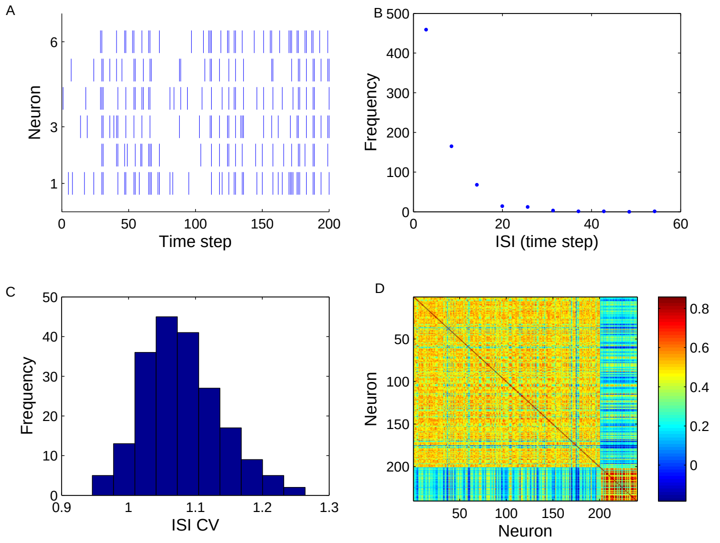

Fig. S5. Neuron activities at 4,000,000th time step. A: spike trains of six randomly selected excitatory neurons during 200 time steps. B: example of an ISI distribution. C: histogram of CV values of a network's excitatory units. D: correlations between all neurons.

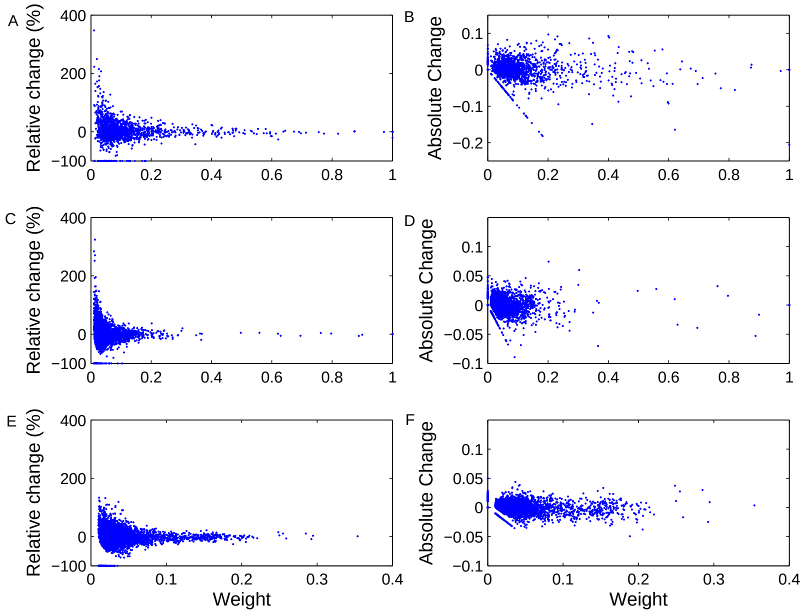

Fig. S6. Synaptic weight fluctuations at different phases. A,B: distribution of relative and absolute synaptic weight changes at 20,000th time step within 3000 time steps respectively. C,D: same as A,B but at 500,000th time step. E,F: same as A,B but at 4,000,000th time step.

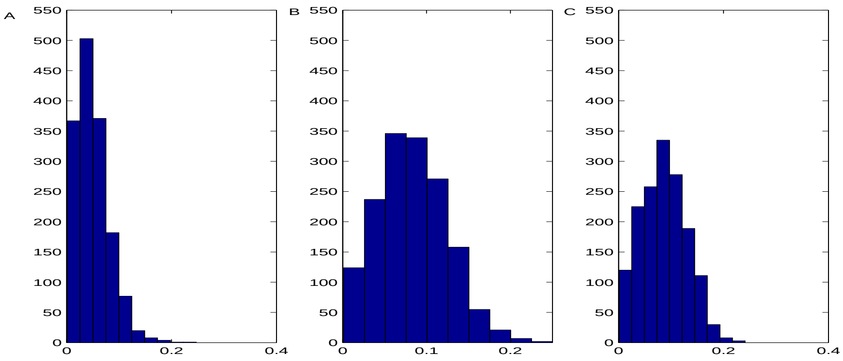

Fig. S7. Distribution of W^{EI} at different phases. A: decay phase. B: growth phase. C: stable phase.

Example 2: Network with 800 excitatory neurons

As an example, Figure S8 plots simulation results of a network with 800 excitatory neurons at decay phase. All other parameters were identical to those used in the main text. For simplicity, we only plot the figure in decay phase.

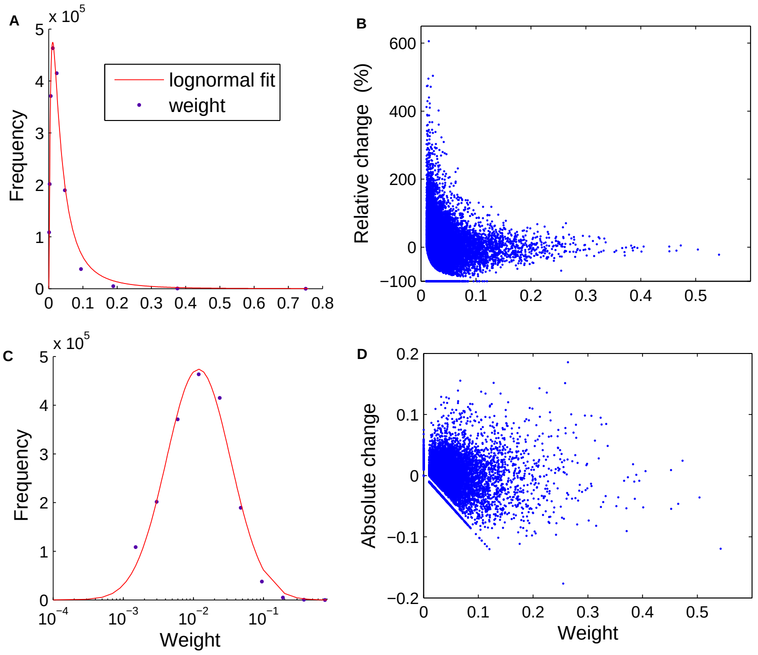

Fig. S8. Results obtained from an 800-excitatory-neuron network. A: distribution of synaptic weight and lognormal fit ($p[w] = 9785\exp[-(\ln[w] + 3.323)^2/(2 \times 1.056^2)]/w$). B: relative synaptic weight changes in 3000 steps. C: same as A but plotted with logarithmic scale on X-axis. D: absolute synaptic weight changes.
